# Supplementary material for: Barley Leaf Ameliorates Citrobacter rodentium-Induced Colitis through Preventive Effects
Source: Nutrients. 2022 Sep 16;14(18):3833. doi: 10.3390/nu14183833 (PMC9502111; doi:10.3390/nu14183833)
Supplement: Supplementary file 1 [file nutrients-14-03833-s001.zip › nutrients-1912278-supplementary.pdf]

## Supplemental file

# Barley leaf ameliorates *Citrobacter rodentium*-induced colitis mainly through preventive effects

Yu Feng, Daotong Li, Chen Ma, Meiling Tian, Xiaosong Hu, Fang Chen*

**This file includes:**

Figure S1. Daily food consumption (A) and water intake (B) of each group;

Figure S2. (A) Representative H&E staining images of cecum tissues. Scale bar, 100 μm. (B) Histopathology score of cecal section

Figure S3. Taxonomic distributions of gut bacterial composition at the genus level;

Table S1. The nutritional composition of barley leaf.

Table S2. The macronutrient composition of chow diet (CD) and an isocaloric barley leaf-(BL) supplemented diet.

**Correspondence:** College of Food Science and Nutritional Engineering, China Agricultural University, No.17, QinghuaEast Road, Haidian District, Beijing100083, China.

**E-mail:** chenfangch@sina.com; **Tel./Fax:** +86-10-62737654 (ext 18).

# Figure S1

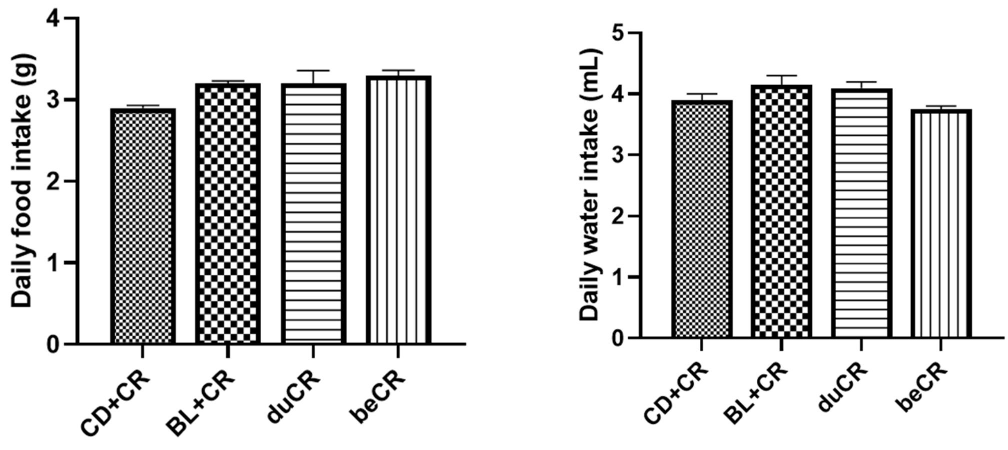


(**A**) (**B**)

**Figure S1 Daily food consumption (A) and water intake (B) of each group.** Mice were fed with a standard chow diet (CD) or an isocaloric BL-supplemented diet for 3 weeks prior to the infection with *Citrobacter rodentium* (CR) for 10 days. (n = 6 per group). (A) Daily water intake and (B) daily food intake of CD- and BL-fed mice of each group. Data are mean ± SEM. a, b means in the same bar without a common letter differ at $P < 0.05$.

# Figure S2

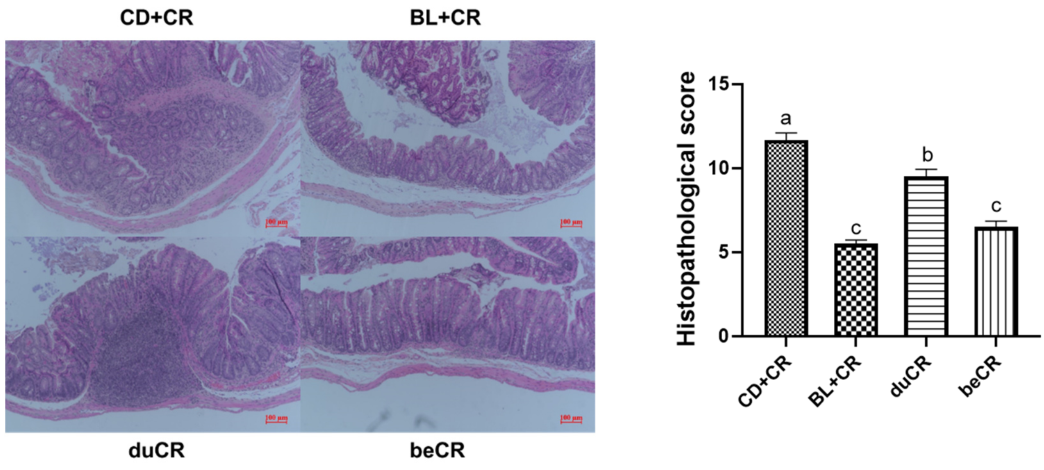


(**A**) (**B**)

**Figure S2 Effect of the beCR and duCR intervention on improving cecal histopathology.** (**A**) Representative H&E staining images of cecum tissues. Scale bar, 100 µm. (**B**) Histopathology score of cecal section; All values are the mean ± SEM, n = 6 if not specified. a, b, c means in the same bar without a common letter differ at *P* < 0.05.

# Figure S3

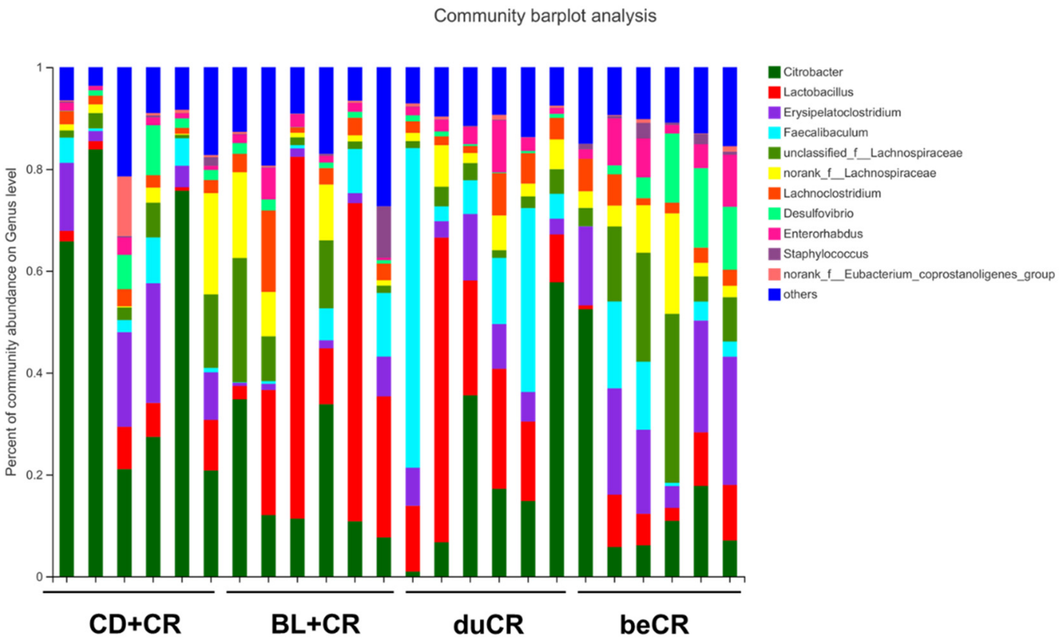


**Figure S3 Effect of beCR and duCR treatment on regulating gut bacterial composition at the genus level.** Each bar represents the taxonomic composition and relative abundance of an individual mouse at the genus level.

# Table S1

**Table S1.** The nutritional composition of barley leaf.

| | **Unit (g/100g)** |
|---|---|
| Protein | 23.9 |
| Fat | 2.98 |
| Carbohydrate | 62.3 |
| Soluble dietary fiber | < 0.01 |
| Insoluble dietary fiber | 53.6 |
| Moisture | 3.99 |
| Ash | 6.8 |

# Table S2

**Table S2.** The macronutrient composition of chow diet (CD) and an isocaloric barley leaf-(BL) supplemented diet.

| **Ingredients (g/kg of diet)** | **Diets** | |
|---|---|---|
| | **CD** | **BL** |
| BL | 0 | 25 |
| Casein | 189.58 | 178.58 |
| L-Cysteine | 2.84 | 2.84 |
| Corn Starch | 298.59 | 280.59 |
| Maltodextrin | 33.18 | 33.18 |
| Sucrose | 331.77 | 327.77 |
| Cellulose | 47.40 | 47.40 |
| Soybean oil | 23.70 | 23.70 |
| Lard | 18.96 | 18.96 |
| Mineral Mix M1002 | 9.48 | 9.48 |
| DiCalcium Phosphate | 12.32 | 12.32 |
| Calcium Carbonate | 5.21 | 5.21 |
| Potassium Citrate | 15.64 | 15.64 |
| Vitamin mix V10001 | 9.48 | 9.48 |
| Choline Bitartrate | 1.90 | 1.90 |
| Total | 1000 | 1000 |
